# The Assessment of Iodine Concentrations in Colostrum and Breast Milk Using ICP-MS: The Impact of Delivery Type, Thyroid Function and Gestational Diabetes—A Pilot Study

**DOI:** 10.3390/foods13142241

**Published:** 2024-07-16

**Authors:** Jadwiga Kryczyk-Kozioł, Paulina Moniak, Paweł Zagrodzki, Ryszard Lauterbach, Hubert Huras, Magdalena Staśkiewicz, Mirosław Krośniak, Paweł Paśko, Robert Podsiadły, Justyna Dobrowolska-Iwanek

**Affiliations:** 1 Department of Food Chemistry and Nutrition, Faculty of Pharmacy, Jagiellonian University Medical College, Medyczna 9, 30-688 Kraków, Poland; jadwiga.kryczyk@uj.edu.pl (J.K.-K.); paulina.moniak@student.uj.edu.pl (P.M.); pawel.zagrodzki@uj.edu.pl (P.Z.); p.pasko@uj.edu.pl (P.P.); 2 Department of Neonatology, Jagiellonian University Hospital, Kopernika 23, 31-501 Kraków, Poland; ryszard.lauterbach@uj.edu.pl; 3 Department of Obstetrics and Perinatology, Jagiellonian University Hospital, Kopernika 23, 31-501 Kraków, Poland; hubert.huras@uj.edu.pl (H.H.); magorecka@su.krakow.pl (M.S.); 4 Institute of Physics, Faculty of Natural Sciences, Jan Kochanowski University, Uniwersytecka 7, 25-406 Kielce, Poland; rpodsiadly@ujk.edu.pl

**Keywords:** iodine, colostrum, breast milk, thyroid, ICP-MS, infants

## Abstract

Considering the spectrum of benefits of breast milk feeding, determining the essential components of an infant’s only food-mother’s milk-seems justified, especially in the case of those whose deficiency (e.g., iodine) may result in developmental disorders. The main aim of this study was the determination of the total iodine content of breast milk (including colostrum and mature milk). A secondary objective was to assess the influence of factors such as the type of delivery, hypothyroidism, gestational diabetes or the stage of lactation on this parameter. The study materials were colostrum and milk after 1 (*n* = 14), 2 and 3 months (*n* = 8) of lactation with a range of iodine concentrations (µg/L): 195–1648 and 170–842, 174–650 and 273–751, respectively. Iodine was determined using the inductively coupled plasma mass spectrometry (ICP-MS). Multivariate statistical analysis revealed, e.g., that delivery by caesarean section or dose of L-thyroxine taken by women to normalise thyroid hormones, had a significant effect on iodine concentrations in breast milk. Further research aimed at assessing the quality of breast milk should also include determining the factors influencing it.

## 1. Introduction

Iodine is an essential substrate in the synthesis of thyroid hormones through which it affects, among other things, growth and development processes, with particular emphasis on the brain [1,2]-crucial from an infant’s development point of view. Furthermore, dynamics of thyroid hormone synthesis in the first weeks of life are specific, i.e., thyroxine (T4) is secreted in a several times higher concentrations per kg of body weight, as in adults [3,4]. Therefore, the thyroid gland in the early stages of postnatal life is extremely sensitive to inadequate iodine supply. Nevertheless, setting precise iodine intake guidelines for infants, especially during the first 6 months of life, is still a challenge. In Poland, the adequate intake (AI) of iodine for infants aged 0–6 and 7–11 months was set as 110 and 130 μg/day, respectively [5], while the European Food Society Authority (EFSA) indicates 70 μg/day but only for infants aged 7–11 months [6]. In turn, the World Health Organisation (WHO) has established a daily recommended iodine intake as 90 μg/day for children under 2 years of age [7].

The benefits of breastfeeding extend beyond fulfilling the nutritional needs of young organism, setting it up for optimal growth and development, influencing, among other things: not only gut microbiota and decreasing the risk of infections (including respiratory infections) or obesity [8] but also cognitive and emotional development [9]. Hence, WHO recommends that infants should be exclusively breastfed during the first 6 months of life [10].

The problem of insufficient iodine supply is still present in the European population, including Poland; therefore, monitoring of intake of this element is needed, especially in groups particularly vulnerable to consequences of iodine deficiency [11,12]. Undoubtedly, infants belong to such a group. Urine is a useful biological material for assessing iodine supply, as approximately 90% of it is removed by the urinary system. Therefore, ioduria (UIC) is widely used as a marker of iodine resources in the human body [13]. However, collecting urine samples, especially among the youngest children, can be challenging; hence, assessing iodine supply via the determination of breast milk iodine concentration (BMIC) seems valuable, particularly as a linear dependence with UIC has been reported [4]. However, it should be emphasised that the production of breast milk undergoes changes. Colostrum (initial milk) is secreted about 6 days postpartum, then transitional milk, and approximately 15 days after delivery, the production of mature milk begins [14]. Thus, the stage of lactation may affect the iodine concentration in milk [15].

Considering both the risk of inadequate, i.e., too low, iodine supply in the population of pregnant women [11] and growing enthusiasm for breastfeeding [16], research into the assessment of various aspects of the quality of the only food of infants-breast milk-is fully justified. In view of the extent of consequences of iodine deficiency during the first months of life, the primary goal of this study was to determine the total iodine content of breast milk, including in different stages of lactation. Iodine concentration in colostrum and milk samples after the mineralisation process was determined using coupled plasma mass spectrometry (ICP-MS), as it is a more preferred analytical method than the Sandell-Kolthoff reaction due to the fact that it is a biological material with a complex matrix [17,18]. In addition, an effort has also been made to identify factors that may affect iodine concentrations in breast milk.

## 2. Materials and Methods

### 2.1. Chemicals

Ultrapure water (18 MΩ cm) was obtained from the Milli Ro & Q water purification unit (Merck-Millipore, Burlington, MA, USA). A 25% aqueous solution of tetramethylammonium hydroxide (TMAH) and potassium iodide was obtained from Sigma-Aldrich (Steinheim, Germany) and tellurium standard solution (1000 mg/L) from Merck (Darmstadt, Germany). A certified reference material (CRM), skimmed milk powder (ERM-BD151, Brussels, Belgium) with iodine concentration of 1.78 ± 0.14 µg/g was used to determine the accuracy of the method.

### 2.2. Study Group Design and Sample Collection

Colostrum samples were taken from 28 women who gave birth at the Jagiellonian University Hospital in Krakow. Milk after 1, 2 and 3 months of lactation was also obtained from 8 participants. They ranged from 20 to 42 years, 155 to 179 cm height and body weight (at birth) 65–103 kg. The study was conducted in accordance with the Declaration of Helsinki and approved by the Ethical Committee of Jagiellonian University (No. 1072.6120.141.2023). Female volunteers enrolled in the study met the following inclusion criteria: over 18 years of age, physiological pregnancy and birth at term (≥37 weeks of pregnancy), singleton pregnancy, no genetic disorders of the foetus, declaration of breastfeeding for at least 3 months of newborns’ life and informed consent of the patient. Exclusion criteria for the study were as follows: the use of an elimination diet due to a medical indication (e.g., patients with phenylketonuria) or for other reasons (e.g., vegans, vegetarians), cancer diagnosis, urinary and genital tract infections in the previous 2 months, multiple pregnancies, use of assisted reproductive technology, active smokers, no compliance to the study protocol and lack of informed consent by the patient. Among the women who participated in the study, half gave birth by caesarean section and half by vaginal section. The number of women who received epidural anaesthesia in our study was 21. However, for none of them was the injection site for epidural anaesthesia disinfected with povidone-iodine (PI), so this procedure did not affect the results. Moreover, 15 participants were diagnosed with gestational diabetes mellitus (GDM), and 14 were diagnosed with hypothyroidism. During the course of the pregnancy, these women were under the medical care of diabetologists and endocrinologists, respectively, and received tailored treatments and followed specific dietary plans. After collection, the biological material was immediately frozen at −21 °C.

### 2.3. Samples Preparation

CRM powder was reconstituted with water according to the manufacturer’s recommendations. Sample preparation was performed according to the method described in [17] with some modifications. Briefly, the thawed colostrum/milk samples and CRM solution were shaken to obtain a homogeneous solution. An amount of 300 μL of biological material was transferred into plastic tubes and tightly twisted. An amount of 0.1 mL of 25% TMAH solution was added to 300 μL of samples and heated in a water bath (WB 22, MEMMERT, Schwabach, Germany) for 3 h at 90 °C. In the next step, the samples were cooled at room temperature. Then, 50 μL of tellurium solution (1 mg/L) was added as an internal standard to each sample and 9.55 mL of milli-Q water. Some milk mineralizates required additional dilution to achieve concentrations within the range of concentrations of the standards used to prepare the calibration curve.

### 2.4. Instrumentation and Conditions

ICP-MS system (Perkin-Elmer SCIEX, ELAN DRC-e, Foster, CA, USA) equipped with Scott spray chamber and glass nebuliser, a quartz torch with a quartz injector tube, was used. ELAN DRC-e was equipped with nickel sampler and skimmer cones. The apparatus parameters are included in Table 1.

### 2.5. Validation Parameters

Before analysing the colostrum and milk samples collected from the volunteers, the analytical method used was validated according to ICH Q2 (R2) guidelines [19]. For calibration purposes, linearity was tested by analysing a sequence of solutions with increasing iodine concentrations: 1.25, 2.5, 5.0, 10.0, 25.0, 50.0 and 100 μg/L. The linearity range was assessed by plotting the analytical signal of iodine against concentration and calculating the coefficient of determination (R^2^) using the method of least squares. An R^2^ of 0.998 indicated that the method was characterised by linearity of the readings over the range of standard concentrations. In turn, the results of the analysis of the milk reference material revealed that the method used was accurate, as the iodine concentration determined (1.65 ± 0.02 µg/g) was within the reference concentration range specified by the manufacturer (1.78 ± 0.14 µg/g). To examine the method precision, 6 samples that were prepared independently (underwent separate mineralisation processes) from the same portion of milk were successively analysed. The method’s precision expressed by the coefficient of variation (CV%) was 1.5%. The limit of detection (LOD) and limit of quantification (LOQ) were expressed as LOD = 3.3 σ/S, LOQ = 10 σ /S, respectively, where σ means the standard deviation of the response and S the slope of the calibration curve. Slope was determined from the regression line of the analyte and σ was calculated from the results of the analysis of 5 blank samples. The obtained limit of detection was 0.38 µg/L, and the limit of quantification was 1.25 µg/L. The determined values of the validation parameters indicated that the method was reliable and can be used for the analysis of colostrum and milk obtained from volunteers.

### 2.6. Statistical Approach

Descriptive statistics (means, confidence intervals of the means, medians, lower and upper quartiles, min and max values) were calculated for iodine milk concentrations in various study groups and subgroups. In the case of means, data were transformed into logarithms and retransformed after calculation because the concentration of iodine in milk had a non-Gaussian distribution. Therefore, the data were shown as means and confidence intervals. The differences between groups were assessed using Kruskal-Wallis (with a post hoc Dunn test) or Mann-Whitney U tests. Changes in iodine concentration throughout the whole experiment were examined using Friedman ANOVA, while the differences between iodine in colostrum and in milk from first month were analysed by means of Wilcoxon test. A probability level of *p* < 0.05 was considered to be statistically significant.

To quantify any dependencies between the investigated parameters, a statistical correspondence analysis (CA) method was used. The nominal data from the questionnaire (dichotomous: no hypothyroidism/presence of hypothyroidism; no gestational diabetes mellitus/presence of gestational diabetes mellitus; delivery by caesarean section/vaginal delivery; multilevel categorical parameter: no L-thyroxine (L-T4) use, L-thyroxine in a dose of less than 50 µg/day, L-thyroxine at a dose of 50 to 100 µg/day, L-thyroxine at a dose of more than 150 µg/day) were gathered in a contingency table together with the iodine milk concentrations transformed into an ordinal scale (four categories) according to the division determined by subsequent quartiles. All these parameters created multidimensional space of original scores (original set of parameters). The analyses of coordinates of these parameters in the new coordinate system of the CA model, generated in the reduced (two-dimensional) space, allowed us to reveal the structure of associations between parameters. In this work, CA model (CAM) was constructed under the condition that its first two dimensions should explain at least 50% of the total inertia in the original set of parameters. Thus, the parameters or their categories with the lowest quality of representation were subsequently discarded as well as associations in the CAM depending only on single coincidence. Those parameters with large absolute values of their coordinates (>0.3) in the CAM were assumed to determine the axes of the new coordinate to the greatest extent. To express the strength of bivariate associations, for the pairs of associated parameters, the cosine of the corresponding angle was calculated using algorithm in Excel created by one of us (RP). The “corresponding angle” was defined as the angle determined by two lines connecting the origin with coordinates of both parameters on the CAM coordinates plot.

Statistical analyses were carried out using Graph Pad Prism v.8.0.1 (GraphPad Software, Boston, MA, USA) and STATISTICA v. 13.3. package (TIBCO Software Inc., Palo Alto, CA, USA).

## 3. Results

The results of iodine determinations in the milk of in the entire group and in individual subgroups distinguished due to various characteristics were summarised in Table 2. Outcomes were expressed as mean and standard deviation or mean and confidence interval, median and range of values (minimum-maximum) and lower and upper quartile.

The iodine concentration determined in the participants’ milk was gaining values over a wide range i.e., 195–2334 µg/L, with a median of 583 µg/L.

In the course of compiling the results, it was examined whether there was a significant dependence between type of delivery and iodine concentration in the women’s colostrum. The mean iodine concentration in colostrum of women who gave birth by caesarean section was significantly higher than in women who gave birth vaginally (*p* < 0.0001). In contrast, the presence of a disease such as hypothyroidism or gestational diabetes mellitus had no significant effect on this parameter. The mean iodine concentration in colostrum of women with hypothyroidism was only slightly lower than that of healthy women (*p* = 0.358). At the same time, it should be mentioned that all participants with thyroid dysfunction took L-thyroxine, but the dose of the drug was not correlated with the iodine concentration in the mothers’ colostrum as well (*p* = 0.611). In the group with and without gestational diabetes mellitus, the means were very similar (*p* = 0.358). However, a statistically significant difference occurred between younger and older women. In the latter group, the concentration of iodine in milk was much higher, but their frequency of caesarean section was also completely different (OR = 32.5, 95% CI 3.1–337.8).

**Figure 1:**
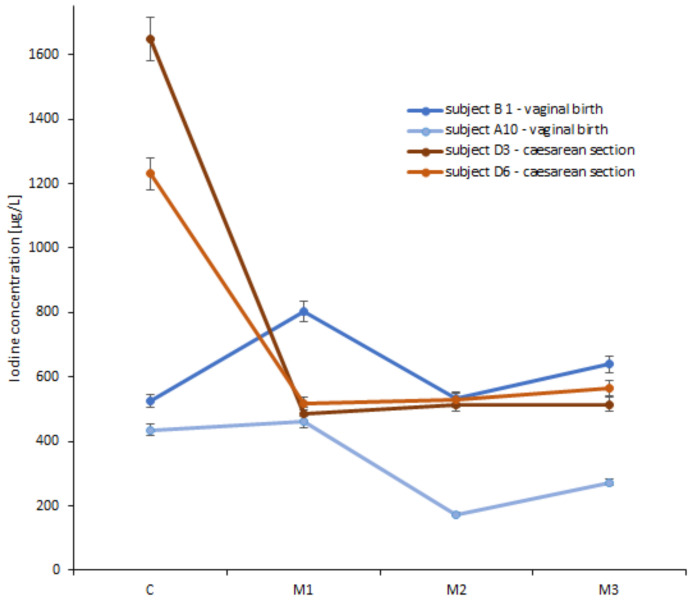
The iodine concentration (µg/L) in colostrum (C) and milk samples collected from selected women over the course of the first three months of lactation (M1–M3), categorised by the mode of delivery: vaginally birth (*n* = 2; B1, A10) and caesarean section (*n* = 2; D3, D6).

Colostrum samples were taken between 1 and 4 days postpartum; however, this had no significant effect on iodine concentrations (*p* = 0.517). Of all the women in the study, 14 of them gave additional sample of milk after 1 month of lactation and 8 of them after a further 2 months. It was observed that the high iodine concentrations that were determined in some colostrum samples (i.e., >1000 µg/L) did not persist in subsequent months of lactation (i.e., <600 µg/L). Example results for selected participants were shown in Figure 1. Nevertheless, no statistically significant differences were observed among iodine concentrations in the milk of women taken in subsequent months of lactation (*p* > 0.05).

### Correspondence Analysis Model

**Figure 2:**
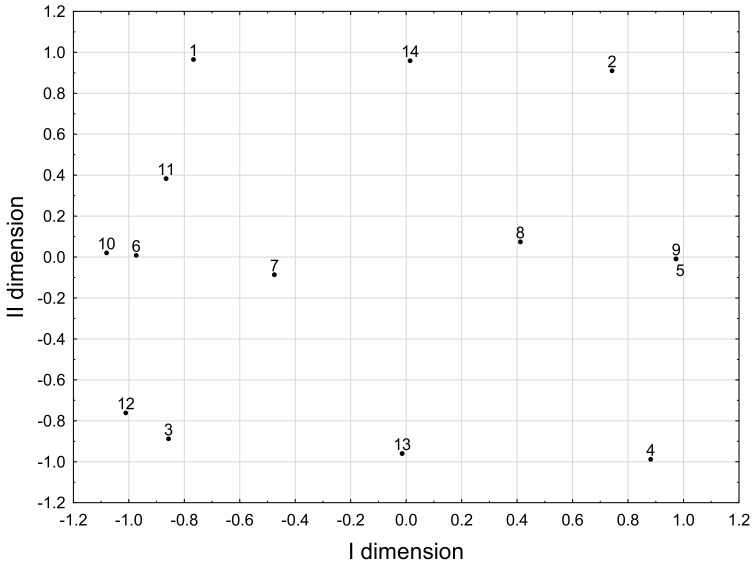
The coordinate values of the tested parameters in the system of the first two dimensions of CAM (meaning of symbols: 1—iodine concentration-quartile 1; 2—iodine concentration-quartile 2; 3—iodine concentration-quartile 3; 4—iodine concentration-quartile 4; 5—no hypothyroidism; 6—hypothyroidism; 7—no gestational diabetes mellitus; 8—gestational diabetes mellitus; 9—no L-thyroxine use; 10—L-thyroxine in a dose of less than 50 µg/day; 11—L-thyroxine at a dose of 50 to 100 µg/day; 12—L-thyroxine at a dose of more than 150 µg/day; 13—delivery by caesarean section; 14—vaginal delivery).

The results from the Correspondence Analysis Model were summarised in Figure 2 and Table 3. CAM concerned the following parameters: (i) iodine concentrations classified in appropriate categories (1–4), determined by the values of subsequent quartiles; (ii) diagnosed thyroid diseases; (iii) diagnosed gestational diabetes mellitus; (iv) the doses of L-thyroxine taken and (v) type of delivery. The model explained 51.8% of the total inertia in the original set of parameters. First, a new dimension was positively loaded mainly by two strongly correlated parameters, ‘no hypothyroidism’ and ‘no L-thyroxine use’ and ‘iodine concentration in quartile 4’, while negatively loaded by two pairs of strongly correlated parameters, ‘L-thyroxine in a dose of less than 50 µg/day and ‘presence of hypothyroidism’, ‘L-thyroxine at a dose of more than 150 µg/day’ and ‘iodine concentration in quartile 3’. Second, a new dimension was positively loaded mainly by three parameters: ‘iodine concentration in quartile 1’, ‘iodine concentration in quartile 2’ and ‘vaginal delivery’. Three other parameters mainly loaded this dimension negatively: iodine concentration in quartile 3’, ‘iodine concentration in quartile 4’ and ‘caesarean delivery’.

## 4. Discussion

Apart from the concept of “fetal programming”, another one, i.e., “first 1000 days of life”, is equally important in the context of harnessing the potential of nutritional factors to increase the child’s chance of optimal growth and development [2]. As already mentioned, breast milk is undoubtedly the best food for infants during the initial few months of life. Data on iodine resources in the European population are worrisome [11], and even worse, lactating women and infants are groups particularly at risk of iodine deficiency [18,20]-one of the key elements for proper child neurodevelopment [2].

Dror et al. (2018), based on a systematic review, indicated an iodine concentration in breast milk of about 150 μg/L to be sufficient for infants (during the first 6 months of life) [18], while Dold et al. (2017) proposed a reference range of 60–465 μg/kg in exclusively breastfeeding women from the iodine-sufficient population [21]; however, the present state of knowledge is not sufficient to indicate the optimal range for this. Therefore, research aimed at determining the iodine content of a child’s first food-breast milk-is extremely relevant, but it is equally important to identify factors that may impact it. This study responds to these needs.

The median (range) of the level of iodine noted for all breast milk samples was equal to 583 (195–2334) µg/L (Table 2). Liu et al. (2022), based on a systematic review of 51 studies, indicated a narrower range of BMIC, i.e., 18–1153 μg/L, and after adjusting for women’s iodine status, the values were as follows: 26–185 μg/L and 15–1006 μg/L in iodine-deficient and iodine-sufficient groups, respectively [22]. The results of studies conducted in the European population also showed lower values [median (lower-upper quartile)], i.e., 148 (98–206) μg/L (Spain) in the group that contained both women taking and not taking iodine supplements during pregnancy (no information on dose) [23] and 112 (80–154) μg/L (Denmark) in the group of women with an intake of iodine at a dose of 150–175 µg/day throughout this period [24]. However, it should be noted that in these populations, there are no official recommendations for iodine supplementation in the pregnant group (following [25,26]). In contrast, in our study, all participants, declared a regular intake of supplements during pregnancy, in which the iodine dose ranged from 150 to 200 µg/day and is consistent with the recommendations of the Polish Society of Gynaecologists and Obstetricians [27]. Hence, it can be assumed that it could have had a beneficial effect on their body’s iodine resources in the perinatal period.

The stage of lactation can also have an impact on BMCI. Nazeri et al. (2018), in a meta-analysis, reported a higher iodine concentration in colostrum compared to mature milk in groups of women from both iodine-sufficient (152.0 μg/L (CI: 106.2–198.7) vs. 71.5 μg/L (CI: 51.0–92.0)) and iodine-deficient (57.8 μg/L (CI: 41.4–74.1) vs. 28.0 μg/L (CI: 13.8–69.9)) regions [28]. In the present study, there were no significant differences in iodine concentration between colostrum and mature milk, although it is worth noting that for colostrum samples, the range was wider (Table 2). Perhaps the lack of statistical differences was due to the small number of samples. Importantly, we also observed that the iodine level in colostrum collected on the first day after childbirth was higher than in colostrum samples collected on days 2–4 as well as in milk samples collected between 1 and 3 months postpartum. In addition, there was a statistically higher BMCI in group of women with delivery by caesarean section compared to those with a vaginal delivery (Table 2), and the CAM model also revealed a positive correlation between the type of birth and iodine concentration, i.e., delivery by caesarean section with iodine concentrations both from 3rd and 4th quartiles, while vaginal birth with iodine concentrations from 1st and 2nd quartiles (Table 3). Thus, the type of delivery may also be an important factor affecting iodine concentration in breast milk. It is well known that the use of povidone-iodine (PI) as an antiseptic can significantly increase iodine levels in both the blood and milk of lactating women. Newborn infants rely entirely on the iodine content in breast milk or in formula for their iodine intake. To maintain a positive iodine balance, full-term infants need an iodine intake of at least 15 μg/kg/day [29]. The transient high iodine levels in early colostrum, which decrease and stabilise over time, could impact infant health. The sodium-iodide symporter (NIS) facilitates the concentration of iodine into breast milk, and normally, iodine levels peak in colostrum, decline in the following weeks and stabilise after one month in iodine-replete women. However, it should be noted that several other factors influence iodine concentration in breast milk as well, including maternal age, urinary iodine levels, iodine supplementation, smoking, salt iodisation and exposure to inhibitors of NIS, such as perchlorate, cyanogenic glucosides from foods like manioc or tapioca and glucosinolates, especially thiocyanates from brassica vegetables [30]. There is some evidence in the literature that has shown that iodine absorption from mucosal surfaces can significantly affect iodine levels; for instance, preoperative vaginal disinfection with povidone-iodine, daily vaginal douches and mouthwashes have been associated with increased serum and urinary iodine levels. Gosset et al. (2008) found that post-surgical iodine irrigation led to thyrotoxicosis [31]. During the perinatal period, which spans from about 24 weeks of pregnancy to 7–28 days after birth, significant changes occur in thyroid function and iodine metabolism. Excessive iodine exposure from antiseptics or contrast media can disrupt thyroid function, leading to transient neonatal hypothyroidism or hyperthyrotropinemia due to the Wolff-Chaikoff effect. The results of previous studies on the effects of topical iodine application on infants are inconsistent. Dold et al. (2017) and Fuse et al. (2023) reported no significant impact of this on thyroid function in mothers and newborns [21,32]. Additionally, Fuse et al. (2023), found that maternal urinary iodine concentrations (UIC) increased after birth but normalised within a month. The study noted transient increases in iodine levels in maternal serum and milk following caesarean delivery, but no significant changes in maternal or neonatal thyroid-stimulating hormone (TSH) or free thyroxine (fT4) levels [32]. Conversely, Smerdely et al. (1989) observed that urinary iodine excretion significantly increased in iodine-exposed infants, with some experiencing elevated TSH and reduced thyroxine levels, although thyroid function normalised by hospital discharge [33]. This suggests that iodine absorption from topical antiseptics could lead to neonatal hypothyroidism, particularly in low-birth-weight infants. Findik et al. (2014) noted changes in maternal fT3, TSH and urine iodine excretion due to povidone-iodine exposure during caesarean operations, though infant urine iodine levels remained unaffected [34]. Overall, the current state of knowledge is insufficient to establish the effects of iodine from antiseptics on maternal/infant health. Nevertheless, it is worth noting that in our study, the average concentration of iodine in colostrum collected on the 1st day after childbirth was characterised by a higher value than in the following days but without significant differences. One potential explanation is that the exposure to iodine (as an antiseptic) at delivery by caesarean section was so short or the antiseptic contained a relatively low concentration of iodine that it had little effect on iodine metabolism in women organisms and thus infant via colostrum. To the best of our knowledge, this study is the first to report on the impact of topical iodine application on colostrum iodine levels in Poland. It is worth underlining that the results of our analyses show that despite the high concentration of iodine in colostrum, its level later decreases to values similar to those observed in other groups. This suggests that the newborn is exposed to high doses of iodine for a relatively short period, which likely does not result in any physiological disturbances in the child. Therefore, our study provides new and significant data on this topic. Nevertheless, considering the small number of participants in this study, our observations should be interpreted with caution, and this aspect requires further research.

The functional status of a pregnant woman’s thyroid gland has great importance for the developing foetus, in which the synthesis of thyroid hormones does not begin until around the 16th–20th week of pregnancy [35,36]. On the other hand, thyroid disorders are becoming increasingly common and can affect up to 11% of the European population [37]. Therefore, this study also made an attempt to evaluate the effect of maternal hypothyroidism on BMCI. Among participants with hypothyroidism, each of them was treated pharmacologically, i.e., we used L-thyroxine in an individually selected dose. No significant differences in BMCI were noted between hypothyroidism and the healthy group. Similar observations were noted relative to gestational diabetes (Table 2). It should be emphasised that all participating women with mentioned diseases besides specialised obstetricians were under the care of endocrinologists or diabetologists, respectively. Thus, appropriately treated hypothyroidism or diabetes during pregnancy (i.e., proper pharmacotherapy/specific nutrition plans) had no significant effect on the iodine content of breast milk. However, the CAM model revealed a positive correlation between ‘iodine concentration-quartile 3’ and ‘L-thyroxine at a dose of more than 150 µg/day’ as well as a negative correlation between ‘iodine concentration-quartile 4’ and ‘L-thyroxine at a dose of 50 to 100 µg/day’ (Table 3). Therefore, this may raise the question of whether the dose of L-T4, after all, does not affect BCMI, and this undoubtedly requires further in-depth analysis in a larger group of women. Unfortunately, data on the effect of thyroid disease on iodine level in breast milk are missing, and Chen et al. (2018) observed the effect of gestational hypothyroidism on changes only in the composition of whey protein [38].

The multivariate statistical analysis showed that higher breast milk iodine concentrations (i.e., iodine in the 3rd and 4th quartiles) coexisted with caesarean section delivery or higher doses of L-thyroxine taken by women to normalise thyroid hormones. This type of analysis concerns parameters (actually-some of their categories) in a reduced coordinate system, where part of the information is lost but also part of the noise is cut off. Therefore, such an analysis may reveal relationships that are difficult to notice in other approaches.

Additionally, in the present study, statistically higher BMCI was observed in a group of women aged 31–42 compared to younger women, i.e., 20–30 years (Table 2).

It is also worth paying attention to the analytical aspect, which may apply to BCMI and which makes it even more difficult to compare the results of different authors. In the papers published to date on the assessment of iodine content in breast milk, three analytical methods have been used: Sandell-Kolthoff reaction, the inductively coupled plasma mass spectrometer method (ICP-MS), reversed-phase high-performance liquid chromatography (HPLC) [17,22], while the preferred one is only ICP-MS. Although the genetic aspect was not within the scope of the present study, it is also worth mentioning inter-individual variations affecting BCMI, i.e., polymorphisms in SLC5A5 gene affecting iodine transport from blood to milk [39]. This highlights the multiple factors far exceeding the biochemical criteria adopted in our study, which have a recognised impact on iodine levels in milk, besides the breastfeeding woman’s diet.

## 5. Conclusions

In conclusion, research on the quality of breast milk, including iodine content, should be continued considering the benefits associated with breastfeeding. Nevertheless, it is also advisable to focus in these studies on looking for potential factors that may affect milk composition. The type of delivery might influence the iodine level in colostrum, but more research is needed to understand the health consequences of the effect of topical iodine applications as antiseptic during childbirth by caesarean section, both for mothers and infants in the first days of life. In such research, it is necessary to take into account aspects like the surface area of skin treated with iodine compounds, exposure time and the concentration of iodine in the applied preparation. Hypothyroidism or diabetes during pregnancy did not have a significant effect on BMCI if proper pharmacotherapy was used; nevertheless, an evaluation of the effect of the L-T4 dose on milk composition should also be included in future studies.

## Figures and Tables

**Table 1:** System operating parameters for ICP-MS.

Parameter	Analytical Value/Feature
Nebuliser gas flow [L/min.]	0.90
RF power [W]	1100
Plasma gas flow [L/min.]	15.0
Cooling gas flow rate [L/min.]	17
Reaction gas	argon

**Table 2:** The descriptive statistics for iodine concentrations in milk (µg/L) in the entire group and in individual subgroups distinguished due to various characteristics.

Studied Group (Number of Participants)	Mean ± SD ^1^ (95%CI)	Median (min.–max.)	LQ-UQ ^2^
Entire group (*n* = 28)	595 * (314; 1130)	583 (195–2334)	350–956
Natural childbirth group (*n* = 14)	400 ± 139 ^a^ (102; 698)	398 (195–606)	289–526
Caesarean section group (*n* = 14)	942 *^a^ (565; 1571)	956 (295–2334)	731–1377
Hypothyroidism group (*n* = 14)	503 * (253; 998)	589 (195–2334)	295–798
No hypothyroidism group/No use of L-thyroxine (*n* = 14)	705 * (400; 1242)	582 (289–1649)	435–1230
L-thyroxine in a dose of less than 50 µg/day (*n* = 5)	410 (249; 676)	302 (267–810)	295–604
L-thyroxine at a dose of 50 to 100 µg/day (*n* = 6)	490 * (193; 1240)	454 (195–2334)	227–731
L-thyroxine at a dose of more than 150 µg/day (*n* = 3)	758 ± 169 (220; 1296)	798 (573–904)	573–904
Gestational diabetes mellitus (*n* = 15)	618 * (323; 1183)	593 (227–1649)	302–1230
No gestational diabetes mellitus (*n* = 13)	571 * (297; 1097)	573 (195–2334)	397–798
Colostrum collected in the first day (*n* = 4)	795 * (301; 2099)	1140 (195–1649)	550–1513
Colostrum collected in the second day (*n* = 8)	565 ± 183 (143; 987)	572 (267–810)	455–695
Colostrum collected in the third day (*n* = 11)	534 * (249; 1145)	397 (227–2334)	295–1008
Colostrum collected in the fourth day (*n* = 4)	618 * (422; 905)	565 (435–1055)	480–830
Colostrum (*n* = 14)	533 * (270; 1051)	559 (195–1648)	289–1008
Milk after 1 month (*n* = 14)	559 ± 180 (173; 945)	568 (170–842)	485–658
Milk after 2 months (*n* = 8)	495 ± 148 (154; 836)	521 (174–650)	461–586
Milk after 3 months (*n* = 8)	562 ± 139 (241; 883)	560 (273–751)	533–645
Younger participants (20–30 years, *n* = 11)	383 ± 133 ^b^ (90; 676)	302 (227–606)	289–526
Older participants (31–42 years, *n* = 17)	819 *^b^ (454; 1476)	810 (195–2334)	593–1230

^1^ Standard deviation; ^2^ Lower quartile-Upper quartile; * Mean and the confidence interval obtained after converting data to logarithms. Results marked with the same letter in upper index differ significantly; levels of significance *p* < 0.0001.

**Table 3:** The correlation coefficients for the pairs of coexisting parameter categories in CAM (only the pairs of parameters with the highest absolute values of their correlation coefficients were given).

Pairs of Correlated Parameters		Correlation Coefficients
no hypothyroidism	no L-T4 ^1^ use	1
hypothyroidism	L-T4 ^1^ at a dose <50 µg/day	0.999
iodine concentration-Q ^2^ 3	L-T4 ^1^ at a dose >150 µg/day	0.988
hypothyroidism	L-T4 ^1^ at a dose 50–100 µg/day	0.918
hypothyroidism	L-T4 ^1^ at a dose >150 µg/day	0.794
iodine concentration-Q ^2^ 2	vaginal delivery	0.784
iodine concentration-Q ^2^ 1	vaginal delivery	0.773
iodine concentration-Q ^2^ 4	delivery by caesarean section	0.736
iodine concentration-Q ^2^ 3	delivery by caesarean section	0.730
iodine concentration-Q ^2^ 4	L-T4 ^1^ at a dose 50–100 µg/day	−0.911

^1^ L-thyroxine; ^2^ quartile.

## Data Availability

The original contributions presented in the study are included in the article/Supplementary Material, further inquiries can be directed to the corresponding author.

## Supplementary Materials

The following supporting information can be downloaded at https://www.mdpi.com/article/10.3390/foods13142241/s1, Table S1: Column coordinates and contribution to inertia in the CA model.
