# Supplementary material for: The Assessment of Iodine Concentrations in Colostrum and Breast Milk Using ICP-MS: The Impact of Delivery Type, Thyroid Function and Gestational Diabetes—A Pilot Study
Source: Foods. 2024 Jul 16;13(14):2241. doi: 10.3390/foods13142241 (PMC11275260; doi:10.3390/foods13142241)
Supplement: Supplementary file 1 [file foods-13-02241-s001.zip › foods-3081060-supplementary.pdf]

Table S1. Column coordinates and contribution to inertia in the CA model.

Parameter category	No.	Coordinates, dimension I	Coordinates, dimension II	Representation quality	Relative inertia	Absolute contribution, I dimension	Squared correlation with I dimension	Absolute contribution, II dimension	Squared correlation with II dimension
Iodine conc. quartile:1	1	-0.76680	0.964944	0.506367	0.083333	0.053293	0.195995	0.122447	0.310372
Iodine conc. quartile:2	2	0.74226	0.909833	0.459583	0.083333	0.049937	0.183651	0.108860	0.275932
Iodine conc. quartile:3	3	-0.85686	-0.887047	0.507019	0.083333	0.066546	0.244735	0.103476	0.262284
Iodine conc. quartile:4	4	0.88140	-0.987730	0.584156	0.083333	0.070412	0.258953	0.128298	0.325203
Hypothyroidism:0	5	0.97350	-0.008829	0.947790	0.055556	0.171795	0.947712	0.000021	0.000078
Hypothyroidism:1	6	-0.97350	0.008829	0.947790	0.055556	0.171795	0.947712	0.000021	0.000078
Gestational diabetes:0	7	-0.47490	-0.086081	0.201879	0.059524	0.037962	0.195457	0.001810	0.006422
Gestational diabetes:1	8	0.41158	0.074604	0.201879	0.051587	0.032900	0.195457	0.001568	0.006422
L-T4:0	9	0.97350	-0.008829	0.947790	0.055556	0.171795	0.947712	0.000021	0.000078
L-T4:1	10	-1.08036	0.021378	0.253836	0.091270	0.075564	0.253736	0.000043	0.000099
L-T4:2	11	-0.86585	0.382998	0.244468	0.087302	0.058243	0.204462	0.016534	0.040006
L-T4:3	12	-1.01072	-0.760423	0.191975	0.099206	0.039681	0.122586	0.032590	0.069389
Type of birth:0	13	-0.01467	-0.959533	0.920919	0.055556	0.000039	0.000215	0.242156	0.920704
Type of birth:1	14	0.01467	0.959533	0.920919	0.055556	0.000039	0.000215	0.242156	0.920704

Explanations for the table and the method of coding parameters:

1. Representation quality - the square of the distance of a given point from the center of a two-dimensional coordinate system divided by the square of the distance of this point from the center of the original coordinate system; Relative inertia – contribution of parameter category in the whole inertia in original, multidimensional coordinate system; Absolute contribution, I (II) dimension - contribution of parameter category in the inertia of I (II) dimension; Squared correlation with I (II) dimension - contribution of I (II) dimension in the inertia of parameter category.
2. The entire iodine concentration range was divided into quartiles; individual concentrations were assigned a corresponding quartile number; thus "Iodine conc. quartile:1" means, that the given concentration was in the first quartile, etc.;
3. 0 – means no feature, e.g. "hypothyroidism:0" means no hypothyroidism; 1 – means occurrence of given feature
4. L-T4:0 – meaning: no L-thyroxine (L-T4) use; L-T4:1 – meaning: L-thyroxine in a dose of less than 50 μg/day, L-T4:2 – meaning: L-thyroxine at a dose of 50 to 100 μg/day; L-T4:3 – meaning: L-thyroxine at a dose of more than 150 μg/day.
5. Type of birth:0 – meaning: caesarean section; Type of birth:1 – meaning: vaginal delivery.
